# Evaluation of a new method of irrigation and aspiration for removal of ophthalmic viscoelastic device during cataract surgery in a porcine model

**DOI:** 10.1186/1471-2415-14-129

**Published:** 2014-11-07

**Authors:** Arisa Mitani, Takashi Suzuki, Yoshitaka Tasaka, Takahiro Uda, Yukako Hiramatsu, Shiro Kawasaki, Yuichi Ohashi

**Affiliations:** Department of Ophthalmology, Ehime University School of Medicine, Shitsukawa, Toon, Ehime, 791-0295 Japan

**Keywords:** Intraocular lens, Ophthalmic viscoelastic device, Irrigation, Aspiration

## Abstract

**Background:**

To determine if a method for irrigation and aspiration (I/A) during cataract surgery provides effective removal of ophthalmic viscoelastic device (OVD).

**Methods:**

Japanese porcine eyes were used to evaluate I/A performance with Technique 1 (the I/A tip placed on the center of the anterior surface of the IOL), Technique 2 (the I/A tip alternately pressed near the edge of the IOL optic anterior surface on one side and then the other to tilt the IOL back and forth), and Technique 3 (the I/A tip inserted behind the IOL optic, between it and the posterior capsule). Techniques 1 and 2 were compared using the Miyake-Apple posterior view video technique to visualize the flow of irrigation fluid containing triamcinolone acetonide particles behind the IOL. To check the efficacy of OVD removal from behind the IOL for of all three I/A techniques, OVD with fluorescein beads were inserted inside the lens capsule before implantation of the IOL. After each I/A technique, eyes were prepared for Miyake–Apple viewing and pictures of the lens capsule were taken using fluorescent microscopy. Residual fluorescein beads in the capsular bag were analyzed.

**Results:**

Technique 1 resulted in a straight flow of fluid behind the IOL, while Technique 2 resulted in a vortex flow. The average amount of OVD retained inside the capsule after using Technique 2 or 3 was significantly lower than after using Technique 1 (p <0.0001).

**Conclusions:**

Technique 2 proved to remove more effectively fluorescein bead-labelled OVD under the IOL than Technique 1.

**Electronic supplementary material:**

The online version of this article (doi:10.1186/1471-2415-14-129) contains supplementary material, which is available to authorized users.

## Background

The ophthalmic viscoelastic device (OVD) is useful tool in modern cataract surgery. It coats and protects intraocular tissues and crates space. OVD which is used for insertion of the IOL into the lens capsule can be trapped behind the IOL. A common complication of OVDs after cataract surgery is an increase in postoperative intraocular pressure (IOP) because of OVD remaining in the lens capsule or the anterior chamber and obstructing the trabecular meshwork [1–6]. Since IOP spikes could cause the damage of the optic nerve and visual disturbance in the patients with glaucoma, ophthalmic surgeons should avoid IOP spikes after cataract surgery.

Along with IOP spikes, postoperative endophthalmitis is a complication of cataract surgery, and sometimes results in severe visual loss. The entry of external bacterial flora is often the cause of acute postoperative endophthalmitis. External bacterial flora probably enter the anterior chamber through the surgical wound; in fact contamination of the anterior chamber at the end of surgery has been noted to be as high as 5.7% to 21.1% [7–10]. Intraoperative or postoperative contamination of the anterior chamber seems to be the initial step of endophthalmitis. Some reports show that bacteria or the exoskeleton of bacteria are attached to intraocular lenses (IOL) which are explanted after either acute or late onset endophthalmitis [11, 12]. Thus, one possibility is that bacteria contaminate the inside of the lens capsule, adhere to the IOL, and proliferate in the eye even without intraoperative complications. Along with clinical cases, we previously showed that *Enterococcus faecalis* inoculated into the lens capsule could lyse the lens capsule with neutrophils and spread into the posterior segment [13]. Since clearance of aqueous humor in the lens capsule seems to be less, microorganisms could grow and cause endophthalmitis.

Washing and cleaning the inside of the lens capsule at the end of surgery could reduce the incidence of IOP spikes and endophthalmitis. The anterior side of the IOL can be washed very well using the irrigation and aspiration (I/A) handpiece because the tip can easily access the anterior surface of the IOL. However, it is difficult to estimate the effectiveness of the I/A method for washing the inside of the lens capsule behind the IOL. Thus techniques which completely remove OVD and clean behind the IOL are needed. OVDs with different properties have been developed to cope with a variety of clinical situations [14–17]. There are several types of OVDs with different molecular weights and concentrations of sodium hyaluronate. A Cohesive OVD (sodium hyaluronate 1.0%) is often used for insertion of the IOL because it tends to hold together as a mass and is relatively easy to remove at the end of surgery [15, 18]. Auffarth *et al.* have previously described the modified rock’n roll technique, in which circular movements of the I/A tip on the anterior surface of the IOL optic tilt and rock the IOL during I/A [19]. The technique was reported to be efficient in removing high molecular weight OVD (Healon 5) which leaves the eye with greater difficulty, behind the IOL. However rotation of the IOL could stress the lens capsule and zonular fibers. Since cohesive OVDs are easier to leave eye than high molecular weight OVD, rotation of the IOL could be unnecessary for removal of cohesive OVDs. To remove OVD from behind the IOL, insertion of the I/A tip under the IOL is an appropriate method. However it can occasionally induce complications such as aspiration of the lens capsule which can cause a tear. This technique can also be difficult for neophyte cataract surgeons. Thus, a possibly easier, safer and more effective I/A technique should be considered when a cohesive OVD is used. To check safe and effective I/A techniques, it is important to know dynamics of irrigation flow behind IOL during surgery. The porcine eyes were usually used for checking techniques because of similar anatomy of human and easy availability [20–24]. We recently observed the dynamic movements of posterior chamber-associated structures, e.g., the lens capsule, zonular fibers, and anterior hyaloid membrane during cataract surgery using view technique in bisected porcine eyes [24]. Furthermore we confirmed influence of eye bisection to anatomy were minimized [22–24]. Thus porcine model could be useful for checking I/A techniques.

In this study, we compared several I/A techniques. First techniques is that placement of the I/A tip on the anterior optic of the IOL with no further manipulation. That can be easily performed. Second techniques is the modified rock’n roll technique, in which alternately pressing the I/A tip near the edge of the IOL optic anterior surface on one side and then the other to tilt the IOL back and forth without rotation. Third techniques is placement of the I/A tip behind the IOL. We conducted two experiments. One is visualization of irrigation fluid flow behind the IOL during I/A techniques (Experiment 1). Another is checking effectiveness of I/A techniques for removal of fluorescein bead-labelled contaminated OVD (Experiment 2).

## Methods

### Porcine eyes

Twenty-eight porcine eyes were obtained from a local abattoir and were examined with a slit-lamp microscope and used within 24 hours of enucleation (3 eyes for experiment 1 and 25 eyes for experiment 2). Eyes with corneal trauma or other obvious abnormalities were not used.

### I/A technique

**Figure 1:**
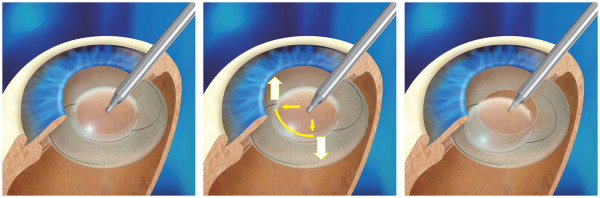
**Removal techniques evaluated in the study.** With Technique 1 (left), the I/A tip was placed on center of the anterior optic of the IOL. Technique 2 (center), alternately pressing the irrigation and aspiration (I/A) tip near the edge of the IOL optic anterior surface on one side and then the other to gently tilt the intraocular lens back and forth. Technique 3 (right), the I/A tip was inserted behind the optic of the IOL.

We compared I/A techniques as follows; placement of the I/A tip on the anterior optic of the IOL with no further manipulation (Technique 1), alternately pressing the I/A tip near the edge of the IOL optic anterior surface on one side and then the other to tilt the IOL back and forth (Technique 2), and placement of the I/A tip behind the IOL (Technique 3) (Figure 1). All techniques used the same settings of 500 mmHg vacuum pressure, 26 mL/min aspiration rate, and bottle height of 60 cm.

### Experiment 1 (visualization of irrigation fluid flow behind the IOL)

Three eyes were prepared using a modified Miyake-Apple method as follows [25–27]. Briefly, each eye was bisected diagonally to the equator using a razor-blade, and the anterior segment was firmly attached to a plastic petri dish with superglue (Aron Alpha, Toagousei Co., LTD. Tokyo, Japan). After making a 2.8 mm corneal incision with a side port incision created at the 10 o’clock position, the anterior chamber was filled with a cohesive OVD (Sodium Hyaluronate 1%, Molecular Weight 1.5 – 3.9 × 10^6^;Opegan Hi®, Santen Pharmaceutical, Osaka, Japan) and a continuous curvilinear capsulorrhexis (CCC) of 5.0 mm was performed. After hydrodissection, the lens nucleus was phacoemulsified using a Phacompo Phacoemulsificator® (Santen Pharmaceutical, Osaka, Japan) using balanced salt solution (BSS) (BSS plus; Alcon, Fort Worth, TX, US) for irrigation. Following phacoemulsification, residual cortical fibers was removed by I/A. And then we used an injector to implant an intraocular lens (Eternity X-60®, Santen Pharmaceutical, Osaka, Japan) into the capsular bag filled with a cohesive OVD. The Eternity X-60® is a monofocal 3-piece spherical hybrid acrylic IOL with a water content of 4.6%. Each IOL (Eternity X-60®, Santen Pharmaceutical, Osaka, Japan) was painted black using a permanent marker before being implanted into porcine eyes. After complete removal of OVD using technique 3, technique 1 or 2 was performed to check dynamics of irrigation flow behind IOL.

To help visualize the irrigation solution, 40 mg/ml triamcinolone acetonide (TA) (Bristol-Myers Squibb Company, New York, US) was added to the BSS. After starting I/A, 0.1 ml of TA was administered for 1 second via an irrigation tube. Dynamics of the irrigation were recorded by a 3CCD camera (DXC-C33, Sony, Tokyo, Japan). Video files, which had 30 frames per second, were converted to picture files using VirtualDub 1.9.11 (GNN General Public License). The whole IOL optic (6mm dia.) was used as the region of interest, and the volume of particles moving under the IOL in each frame was quantified as pixel intensity using image J software (National Institutes of Health, Bethesda, MD, US) [28]. The pixel intensity before irrigation was subtracted from the pixel intensity of each frame.

A grid line was overlaid on each picture frame. We calculated the distance particles moved across a 21 square grid for six successive frames, expressed as a drawing vector. We analyzed both methods in one eye, and repeated the testing in three different eyes.

### Experiment 2 (removal of fluorescein bead-labelled OVD)

Twenty-five porcine eyes were used without dissection. Lensectomy was performed by phacoemulsification and I/A as describe previously. Following exchange to air in the anterior chamber, 0.1 ml of 5% 1.0 μm-fluorescein bead solution (Fluoresbrite™ Carboxylate YG 1.0 micron Microspheres; Polysciences Inc, Pennsylvania, US) was inserted into the lens capsule followed by 0.3 ml of Opegun Hi®. The IOL (Eternity X-60®, Santen Pharmaceutical, Osaka, Japan) was then implanted in the lens capsule. The eyes were then distributed into five groups according to I/A technique, I/A duration, and the location of the I/A tip during OVD removal as shown Table 1. Technique 1 was used in Group A and B, while Technique 3 was used in Group C. Technique 2 was used for Group D and Group E. In Group D, I/A was done for 10 seconds twice per side. In Group E, I/A was done for 5 seconds four times per side.

After the procedure, each eye was cut horizontally at the equatorial region using a razor-blade, and a picture of the lens capsule was taken using fluorescent microscopy (SteREO Lumar V12, Zeiss, Jena, Germany). Using image J software, the amount of residual fluorescein beads under the IOL were measured by pixel count and the results were analyzed [28]. Experiments were performed with five eyes per group.

**Table 1:** **Groups for removal of OVD**

Group	Technique	I/A duration
A	Technique 1	20 sec
B	Technique 1	40 sec
C	Technique 3	20 sec
D	Technique 2	20 sec (5 sec per side ×4)
E	Technique 2	20 sec (10 sec per side ×2)

### Statistical analyses

Data in experiment of fluid dynamics were analyzed by Student’s t-test for significance. Tukey-Kramer tests were used to compare the techniques in the experiment of removal of fluorescein bead-labelled OVD. Values of p <0.05 were considered statistically significant.

## Results

### Experiment 1

**Figure 2:**
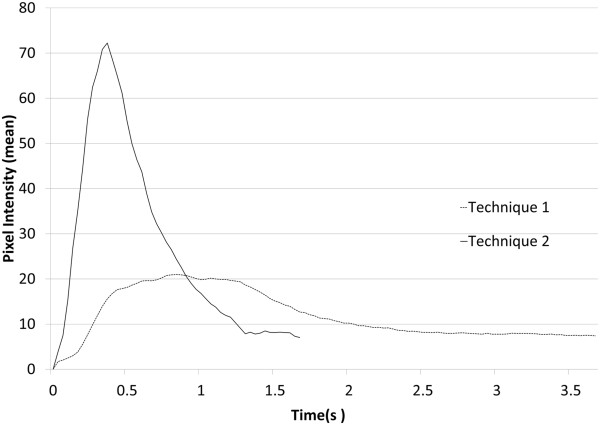
**Histogram of pixel intensity in eye with Technique 1 (dashed line) and Technique 2 (solid line).** Y-axis showing pixel intensity of TA, and X-axis showing time.

**Figure 3:**
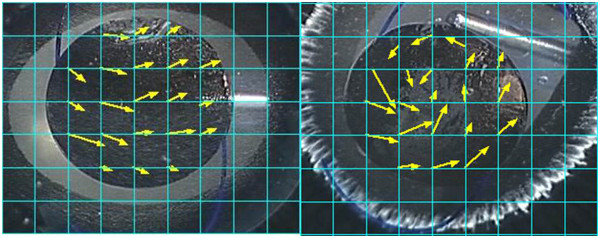
**Movement of particles across a grid during next 0.2 seconds in a porcine eye showing Technique 1 (left) and 2 (right).** The yellow arrows indicate the distance and direction in which particles moved for 0.2 seconds.

Irrigation solutions behind the IOL in all tested eyes were visualized (Additional file 1). We checked flow of irrigation solution using both methods in three different eyes. Since flow pattern of particles in each technique were similar, a representative eye was estimated for movement of particles. Figure 2 shows a histogram of the pixel intensity at each time. We quantified pixel intensity of TA particles behind IOL in technique 1 or 2. The pixel intensity using Technique 2 increased and decreased in a shorter time span compared to Technique 1. To evaluate the dynamic flow of irrigation solution, we measured the distance and direction TA particles traveled for 6 frames (0.2 seconds). Figure 3 shows the vector in which particles crossed the grid lines as yellow arrows. The flow of the irrigation solution using Technique 1 was in one direction at an average distance of 0.66 mm (±0.23 mm) in the 0.2 seconds. For Technique 2 the flow was a focal vortex with an average distance of 0.76 mm (±0.27 mm). There were no statistically significant differences between two methods.

### Experiment 2

**Figure 4:**
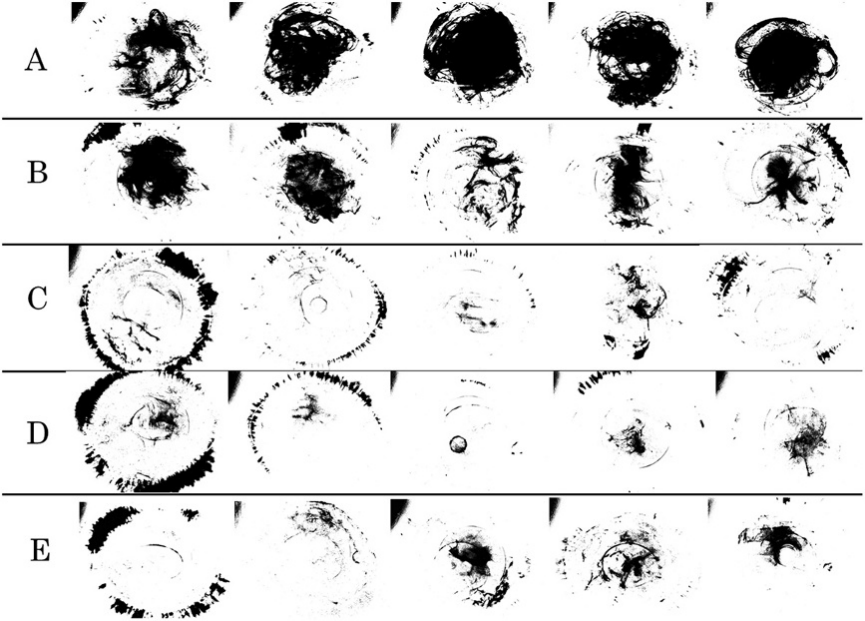
**Photograph of fluorescent pixel after removal of the fluorescein bead-labelled OVD in all five eyes of each group (A, B, C, D,and E).** Black pixel showing residual fluorescein beads.

**Figure 5:**
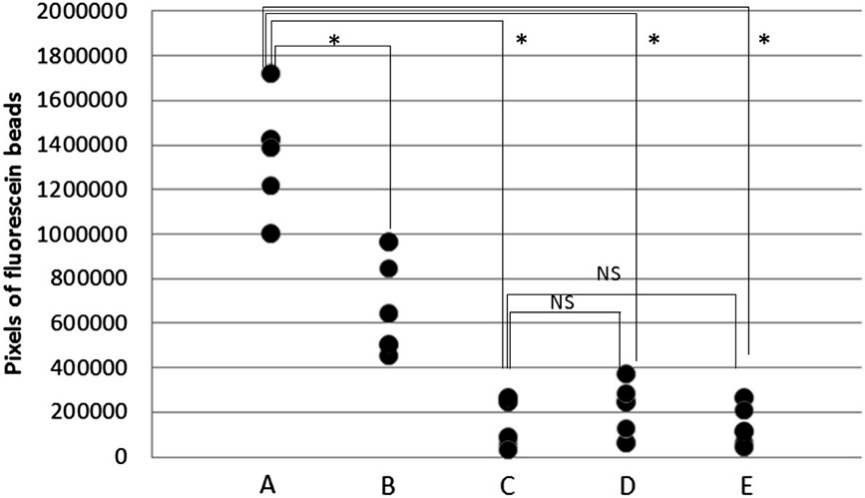
**Pixel intensity after removal of the fluorescein bead-labelled OVD in each group.** *P <0.001, (NS = not significant). Tukey–Kramer multiple comparison test, two-sided. Data represent individual values.

Fluorescent pictures were converted to white and black pictures using image J. Black pixel showed residual fluorescein beads. Figure 4 shows the pixel intensity of residual fluorescein beads for five eyes of each five group. Black pixels were found more in group A than in group B, C, D, or E. The average pixel intensity of fluorescein beads retained inside the capsule in group B, C, D and E was significantly lower than those of Group A (p < 0.001, Turkey-Kramer test) (Figure 5). There were no significant differences in residual fluorescein beads among Group B, Group C, Group D, and Group E.

## Discussion

Removal of OVD from behind the IOL is critical to prevent IOP spikes, avoid shifts in centration of IOL as well as capsular block syndromes, and ensure sterility in the eye after surgery. If OVD behind the IOL has continuity to the anterior chamber, OVD could be removed by the I/A tip on the IOL. However it is difficult to remove OVD once its continuity to anterior chamber is lost. In that case, OVD should be displaced by the flow of irrigation solution and aspirated out of the eye. Although there are some reports which demonstrate irrigation fluid flow in the anterior chamber [21, 29], little is known about irrigation fluid flow behind the IOL during I/A. Kaji *et al.* visualized irrigation fluid flow using 3-dimensional images and demonstrated the flow velocity decreased with increasing distance from the iris plan [21]. In this study, we could observe irrigation fluid flow behind the IOL during I/A using a blackened IOL and Miyake-Apple view method. We could not analyze flow using 3-dimensional images. However 2-dimensional images should suffice because of the minimal distance between the posterior capsule and the IOL. This study demonstrated different patterns of irrigation fluid flow due to the location of the I/A tip. The technique in which the I/A tip is held steady on the center of the anterior surface of the IOL optic appears to provide irrigation fluid flow parallel to the I/A handpiece. In this circumstance, fluid flow from the I/A tip could move to the equator of the lens capsule and repulse to area between IOL and posterior capsule. Technique 2, in which the I/A tip gently pressed down on alternate edges of the IOL optic anterior surface, had a vortex pattern of irrigation flow. The histogram of particle pixel intensity shows that the clearance of particles in this Technique 2 is more rapid than with the I/A tip simply centered on the IOL optic. Thus a vortex pattern of irrigation fluid induces a more rapid exchange of fluid and is therefore more effective in cleaning out the space between the IOL and the posterior capsule.

Along with visualizing and quantifying the flow of irrigation fluid behind the IOL, we also used 1.0 μm-fluorescein beads to determine the amount of residual fluorescein bead-labelled OVD behind the IOL after I/A. Because the diameter of gram positive cocci causing endophthalmitis, such as staphylococci, is about 1 μm, fluorescein beads ought to imitate bacterial contamination, as described previously [23]. This study demonstrated that the amount of residual fluorescein depends on how long the I/A tip is left on the center of the IOL; I/A for 40 seconds removed more beads than only 20 seconds, indicating that it might take longer to remove OVD from behind the IOL if the tip is only placed on the center of the anterior surface of the IOL. This was confirmed by the fact that the amount of residual fluorescence was less after cleaning with the I/A tip inserted between the IOL and posterior capsule. We reasonably conclude that doing I/A with the tip behind the IOL removes OVD more effectively. Importantly, this study demonstrated that technique 2 removed OVD as effectively as using the tip behind the IOL. To effectively remove OVD from behind the IOL, it is considered important to insert the tip behind the IOL. However this technique can induce complications such as aspiration of the lens capsule, causing a tear. It can also be difficult neophyte cataract surgeons to learn and can be difficult for even experienced surgeons in cases where the CCC is small. In contrast, technique 2 should be easy for a beginner to learn. In previous reports, the rock n roll technique in which the I/A tip was moved in quick circular movements on top of the IOL along with Technique 2 could completely remove Healon 5 from the capsular bag [19]. Furthermore ‘Judders’ which are periodic, abrupt, horizontal displacements of the intraocular lens could remove OVD safely and effectively [30]. Thus it is critical to move the IOL for removal of OVD behind the IOL optic.

Some limitations exist in our porcine eye study. First, although the general trends we observed in porcine eyes are probably similar to those in humans, the IOP changes observed in our model may not exactly reflect the changes in human eyes due to the absence of aqueous flow. Second, the anatomic structure of the anterior segment, especially the zonules of Zinn in porcine eyes, are similar to that of human eyes, but the integrity of the tissue may be weakened in an enucleated porcine eye. Thus, further investigations are needed to check irrigation fluid flow behind the IOL during surgery.

Therefore this study demonstrated the importance of specific removal techniques for safe and complete removal of OVDs. Surgeons must be aware of the potential adherence of OVD to the posterior surface of the IOL and pay close attention to its complete removal in order to minimise bacterial contamination and elevated intraocular pressure after surgery.

## Conclusions

This study demonstrated the importance of specific removal techniques for safe and complete removal of OVDs. Alternately pressing the I/A tip near the edge of the IOL optic anterior surface on one side and then the other to gently tilt the IOL back and forth, is an effective method for cleansing behind the IOL and for removing OVD from behind the IOL. Surgeons must be aware of the potential adherence of OVD to the posterior surface of the IOL and pay close attention to its complete removal in order to prevent bacterial contamination and elevated intraocular pressure after surgery.

## Electronic supplementary material

Additional file 1: **Dynamics of irrigation fluid flow behind the IOL.** Visualization of irrigation fluid flow behind the IOL in Technique 1 and 2. The techniques produced a view of the movement of particles during I/A. (MP4 10 MB)
